# Skin manifestations of inborn errors of NF-κB

**DOI:** 10.3389/fped.2022.1098426

**Published:** 2023-01-17

**Authors:** Yitong Shen, Anne P. R. Boulton, Robert L. Yellon, Matthew C. Cook

**Affiliations:** ^1^ Department of Immunology, Cambridge University Hospitals, Cambridge, United Kingdom; ^2^ Centre for Personalised Immunology, Australian National University, Canberra, Australia; ^3^ Cambridge Institute of Therapeutic Immunology and Infectious Disease, and Department of Medicine, University of Cambridge, United Kingdom

**Keywords:** NF-κB, immunodeficiency, genetics, skin, ectoderm abnormalities

## Abstract

More than 400 single gene defects have been identified as inborn errors of immunity, including many arising from genes encoding proteins that affect NF-κB activity. We summarise the skin phenotypes in this subset of disorders and provide an overview of pathogenic mechanisms. NF-κB acts cell-intrinsically in basal epithelial cells during differentiation of skin appendages, influences keratinocyte proliferation and survival, and both responses to and amplification of inflammation, particularly TNF. Skin phenotypes include ectodermal dysplasia, reduction and hyperproliferation of keratinocytes, and aberrant recruitment of inflammatory cells, which often occur in combination. Phenotypes conferred by these rare monogenic syndromes often resemble those observed with more common defects. This includes oral and perineal ulceration and pustular skin disease as occurs with Behcet's disease, hyperkeratosis with microabscess formation similar to psoriasis, and atopic dermatitis. Thus, these genotype-phenotype relations provide diagnostic clues for this subset of IEIs, and also provide insights into mechanisms of more common forms of skin disease.

## 1. Introduction

Mammalian nuclear factor kappa-light-chain-enhancer of activated B cells (NF-κB) comprises a family of homo- and hetero-dimers made of five rel-homology domain-containing proteins (c-Rel, RelA, p105/50, p100/52, and RelB, encoded by *REL, RELA, NFKB1, NFKB2, and RELB*, respectively) (1, 2) (Table 1). Discovery of NF-κB provided the first evidence for tissue-specific transcription based on post-translational regulation of transcription factors in response to cellular stimulation or environmental cues (3). Nuclear translocation of NF-κB influences numerous transcriptional programs critical for activation, differentiation and survival of cells of innate and adaptive immunity, but also for organogenesis of primary and secondary lymphoid organs. Expression of NF-κB is not confined to the immune system. Many non-haemopoietic tissues also rely on NF-κB to maintain homeostasis. This includes the skin and other ectoderm-derived tissues, where NF-κB operates cell-intrinsically to maintain keratinocyte adhesion, homeostasis, and epidermal organisation.

**Table 1:** Summary of NF-κB proteins and their regulators.

Protein	Other name (s)	Gene	Unabbreviated name
**NF-κB family**	** **	** **	**Nuclear factor kappa-light-chain-enhancer of activated B cells**
RelA	p65	*RELA*	
c-Rel		*REL*	
p50		*NFKB1*	
RelB		*RELB*	
p52		*NFKB2*	
**IκB family**	** **	** **	**Inhibitors of NF-κB**
IκBα		*NFKBIA*	Inhibitor of NF-κB, alpha
NF-κB1	p105	*NFKB1*	
NF-κB2	p100	*NFKB2*	
**IKK complex**	** **	** **	**Inhibitor of NF-κB kinase**
IKKα	IKK1	*CHUK*	Inhibitor of NF-κB kinase, subunit alpha (protein); Conserved helix-loop-helix ubiquitous kinase (gene)
IKKβ	IKK2	*IKBKB*	Inhibitor of NF-κB kinase, subunit beta
NEMO	IKKγ	*IKBKG*	NF-κB essential modulator
NIK	* *	*MAP3K14*	NF-κB inducing kinase (protein); Mitogen-activated protein kinase kinase kinase 14 (gene)
**LUBAC**	** **	** **	**Linear ubiquitin chain assembly complex**
HOIP		*RNF31*	HOIL-1 interacting protein (protein); RING finger protein 31 (gene)
HOIL1		*RBCK1*	Heme-oxidized IRP2 ubiquitin ligase-1 (protein); RanBP-type and C3HC4-type zinc finger-containing protein 1 (gene)
SHARPIN		*SHARPIN*	Shank-associated RH domain-interacting protein
**CBM Complex**	** **	** **	**Card-Malt-BCL10 complex**
CARD11	CARMA3	*CARD11*	Caspase recruitment domain-containing protein 11
MALT1		*MALT1*	**Mucosa-associated lymphoid tissue lymphoma protein 1**
BCL10		*BCL10*	B cell lymphoma 10-endoded protein
**TNFR1 signaling complex**	** **	** **	**Tumour necrosis factor receptor 1 signalling complex**
TRADD		*TRADD*	Tumor necrosis factor receptor type 1-associated DEATH domain protein
RIPK1		*RIPK1*	Receptor-interacting serine/threonine-protein kinase 1
RIPK4		*RIPK4*	Receptor interacting serine-threonine kinase 4
TRAF2		*TRAF2*	TNF receptor-associated factor 2
TAK1		*MAP3K7*	Transforming growth factor-beta-activated kinase 1 (protein); Mitogen-activated protein kinase kinase kinase 7 (gene)
TAB2/3		*TAB2*	TGF-beta-activated kinase 1 and MAP3K7-binding protein
cIAP1/2		*CIAP1*	Inhibitor of apoptosis protein 1/2
**Deubiquitins**	** **	** **	** **
A20		*TNFAIP3*	Tumor necrosis factor, alpha-induced protein 3 (gene)
OTULIN		*OTULIN*	OTU domain-containing deubiquitinase with linear linkage specificity
CYLD	* *	*CYLD*	Cytoskeletal-associated protein-glycine-conserved domains

Genetic defects have been identified in NF-κB molecules, their upstream signalling pathways, and regulator components (4–7). Since NF-κB is expressed in both the immune system and cellular components of the skin, it is not surprising that many of the inborn errors of immunity (IEI) arising from defects in NF-κB result in dermatological and mucosal phenotypes. Indeed, these are sometimes the presenting features.

## 2. NF-κB

### 2.1. Overview of NF-κB activation

**Figure 1:**
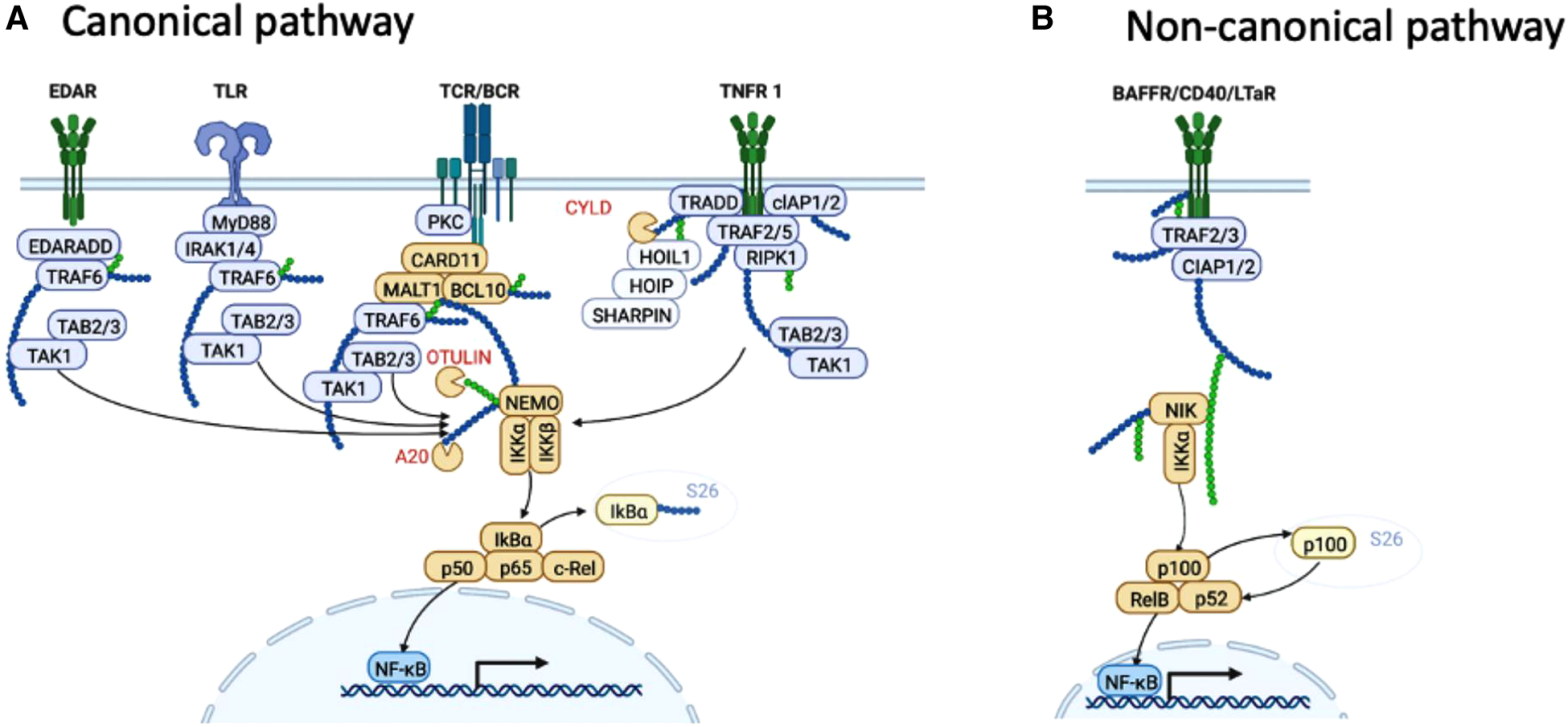
NF-κB pathways. (**A**) Summary of ligands and signalling events that lead to activation of the canonical (classical) NF-κB pathway. (**B**) Summary of ligands and signalling events that lead to activation of the non-canonical (alternative) NF-κB pathway. Proteins discussed in this review are shown in orange. Created with BioRender.com.

Transcriptionally active forms of NF-κB consist of dimers that recognize *cis*-acting κB sites in promoter and enhancer elements (e.g., the decameric sequence 5′-GGGACTTTCC-3′; consensus 5′-GGGPuNNPyPyCC-3) of many genes (8). A defining feature of NF-κB is its rapid response to cues from the cell-environment, which is possible because NF-κB dimers and higher order complexes exist pre-formed and maintained in a latent state within the cytoplasm by a family of inhibitors of NF-κB (IκB), including IκB*α*, IκBβ, IκB*δ* (p100, see below), IκB*ε*, BCL3, and IκBNS, each containing approximately six ankyrin repeats (Table 1) (9–11) NF-κB becomes transcriptionally active when these IκB molecules undergo transient degradation, which is triggered by the IκB kinase (IKK) complex (Figure 1).

NF-κB activation can proceed through either of two pathways determined by specific kinases, referred to as canonical (or classical) and non-canonical (or alternative). Dimers comprising p50 (NF-κB1), RelA and c-Rel become transcriptionally active *via* the canonical pathway, while dimers comprising RelB and p52 become transcriptionally active *via* the non-canonical pathway (12). Tissue-specific regulation of NF-κB results from cell context-specific cues that activate NF-κB *via* various receptors. In the immune system, these include pattern recognition receptors such as toll-like receptors (TLR), NOD-like receptors, and pro-inflammatory cytokine receptors, especially IL-1 and TNF. The canonical pathway can also be activated downstream of B cell and T cell receptors, and in basal epithelial cells, from ligation of ectodysplasin associated receptor (EDAR) (Figure 1). Different membrane-proximal signalling pathways are responsible for delivering the signal after different types of receptor are liganded. Thus, a complex of TRAF6, MyD88 and IRAK1/4 signal from TLRs, protein kinase C (PKC) activates the CARD11-BCL10-MALT1 (CBM) complex downstream of T cell receptor (TCR) and B cell receptor (BCR), and the so-called complex 1, comprising RIPK1, TRADD, cIAP1 and 1, and TAB2 and 3 signal from TNFR1. These membrane proximal events converge on the IKK complex comprising IKKα, IKKβ, and IKKγ (aka NEMO) (13–16), which phosphorylate IκB molecules (17), tagging them for ubiquitination and 26S proteasomal degradation. As a result, NF-κB components p65, c-Rel and p50 are liberated to translocate to the nucleus.

While bearing similarities to canonical activation, the non-canonical pathway differs in kinetics and mechanism of activation, and hinges on p100 processing to p52, which unlike p105 processing, is regulated rather than constitutive (18). NF-κB-inducing kinase (NIK) undergoes constant turnover in resting cells but after appropriate stimulation, NIK turnover is retarded to permit formation of complexes with IKKα, which are responsible for phosphorylation of p100 (NF-κB2). Limited proteasomal degradation of phosphorylated p100 yields p52, which forms transactivating heterodimers with RelB (19).

### 2.2. NF-κB regulation

NF-κB is subject to tight regulation mediated by many layers of feedback. Negative regulation is intrinsic to NF-κB signalling. For example, p100 and p105 which are precursors of active NF-κB transcription factors are themselves members of the IκB family (20), and p50 and p52 homodimers are inhibitory (21). Post-transcriptional regulation by phosphorylation and both K63 and M1 linear ubiquitination affects protein abundance and protein-protein interactions. Linear ubiquitination is mediated by the linear ubiquitin (LUBAC) complex, comprising HOIP, HOIL1 and SHARPIN (22). Deubiquitinases A20, CYLD, and OTULIN are critical regulators of NF-κB activity (23, 24). The deubiquitinating action of A20 has been attributed to the N-terminal OTU domain that removes K63 complexes from NEMO, RIPK1, TRAF6, and MALT1. The action of A20 is complex, however, as C-terminal zinc fingers have E3 ligase activity, and A20 also protects M1 chains (added by HOIP) from removal, which might limit apoptosis after TNF ligation (25). Otulin (OTU deubiquitinase with linear linkage specificity) removes linear polyubiquitin from proteins that had been modified by the LUBAC complex (26).

### 2.3. Actions of NF-κB in the skin

NF-κB mediates organisation and differentiation during development of the ectoderm into skin, its appendages (apocrine glands, hair, and nails), as well as mammary glands, nervous system, placodes, anterior hypophysis, lens, and olfactory epithelium (Table 2) (27, 28). Signals necessary for differentiation of ectodermal progenitors for formation of skin appendages depends on ligation of the TNFR superfamily member EDAR and its downstream adaptor protein EDARADD, which activates canonical NF-κB. Defects in EDAR and EDARADD result in ectodermal dysplasia but as they are skin-specific, no systemic features (27, 29–31). These findings provide important insights into the phenotypes conferred by defects in NF-κB but this review will focus on defects that are associated with concurrent immune defects.

**Table 2:** Ectodermal tissues.

Skin
Hair
Teeth
Mammary glands
Peripheral and central nervous system
Neurogenic placodes
Anterior pituitary
Lens
Olfactory epithelium
Mucosal epithelium
Pigmented cells
Pharyngeal arches (partial)

**Figure 2:**
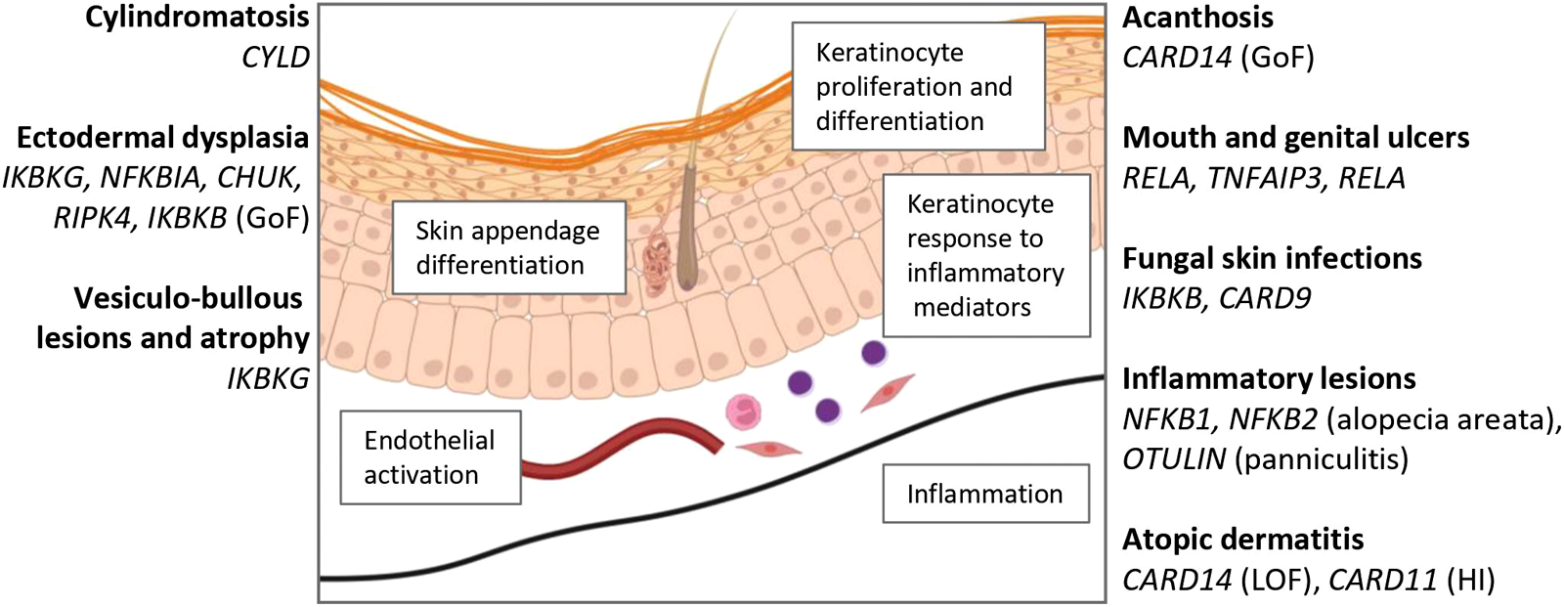
Summary of skin pathology associated with defects in NF-κB. Created with BioRender.com.

In addition to ectodermal development, NF-κB is also important for regulation of keratinocyte growth and differentiation, skin inflammation, and cutaneous immunity (32) (Figure 2). Mouse studies have identified how the interplay of these signals regulates keratinocyte homeostasis. Dominant negative mutation of *Nfkbia* (IκBα) results in neonatal epidermal hyperplasia, which appears to be a cell-intrinsic action in keratinocytes (33, 34). Consistent with these observations, constitutive p65 activity has been found to act cell-intrinsically in basal keratinocytes to inhibit their proliferation (33).

The hypothesis that NF-κB arrests keratinocyte proliferation permitting differentiation was challenged by a mouse model of epidermal keratinocyte-specific IKKβ deficiency, in which mice were born with macroscopically normal skin and developed a cutaneous inflammatory response before progressive dermatitis commencing on day 4–5 (35). In this model, epidermal proliferation was blocked by TNFR deficiency, suggesting that NF-κB regulates the proliferative response of keratinocytes in response to TNFR1 ligation (32, 35).

TNF-dependent inflammation is also a feature of both models in which there is reduction or enhanced NF-κB activity. *Ikbkg* (NEMO) deficiency was shown to arrest keratinocyte growth and trigger dependent inflammation, both of which were TNF-dependent (36). Similarly, *Tak1* deletion, which reduces canonical NF-κB activation also results in TNF-mediated skin inflammation (37). On the other hand, *Tnfaip3* deletion, which enhances NF-κB responses, resulted in marked TNF-dependent epidermal proliferation (38), and keratinocyte apoptosis and dermal TNF-dependent inflammation was observed with keratinocyte-conditional deficiency of *Traf2* (39).

IKKα deficiency identified a further level of complexity. IKKα is critical for normal keratinocyte differentiation, including expression of filaggrin and loricin, as well as for tooth development (40–42), however, this action is mediated independently of its kinase activity and, indeed, NF-κB signalling (43). The action of IKKα nevertheless depends on its nuclear localisation (44). Defects in keratinocytes conferred by *Chuk*^−/−^ (i.e., IKKα-deficient) can be overcome by a soluble factor originally named keratinocyte differentiation factor, but the mechanism of action of IKKα on keratinocyte differentiation remains unclear (43). A potential mechanism is *via* epidermal growth factor receptor (EGFR) signalling, as IKK*α* negatively regulates EGFR *via* ADAM (A disintegrin and metalloprotease) expression in keratinocytes (45).

## 3. Inborn errors of NF-κB and skin pathology

### 3.1. Overview

As outlined below, the spectrum of skin pathology observed in patients with NF-κB defects reflects the requirement for tight regulation of NF-κB. For many genes in the NF-κB pathway, severe phenotypes have been reported for complete loss of function mutations. Components of the NF-κB pathway also appear to be susceptible to the actions of missense mutations, possibly because of the large number of protein-protein interactions required for NF-κB activation. In many instances, phenotypes are also conferred by hypo- and hypermorphic mutations, and these phenotypes often differ significantly from those conferred by loss-of-function (LoF) mutations.

It is important to note that as a consequence of the complex regulatory network for NF-κB, the outcome of a hypermorphic or hypomorphic mutation of a specific protein depends on whether the affected protein is a positive or negative regulator of NF-κB. For example, gain-of-function (GoF) mutation of a negative regulator such as IκBα will have a similar functional consequence as LoF mutation of a IKK complex component such as NEMO (46). Furthermore, as revealed by mutations in p100 (*NFKB2*), a protein with both IκB activity (*via* the p100 precursor) and transcriptional activity (*via* p52), both LoF and GoF can be conferred by the same mutation.

### 3.2. Defects of the IKK complex

#### 3.2.1. *IKBKG* (NEMO)

Loss-of-function (amorphic) mutations in *IKBKG* result in incontinentia pigmenti (IP) (47). IP usually begins shortly after birth. The phenotype is variable but in classic form is characterised by four distinct dermatological stages, beginning with vesiculobullous eruption (Stage I) then verrucae (Stage II), hyperpigmentation (Stage III), and finally atrophic hypopigmentation (Stage IV) (48, 49). Mucocutaneous ulcers of both mouth and perineum are also common, and have resulted in the concurrent diagnosis of Behcet's disease and IP (50, 51). Now that similar manifestations have been observed with *TNFAIP3* haploinsufficiency (see below), it seems likely that these manifestations reflect the action of NEMO on A20 ubiquitination (52). In 30%–40% of cases, the molecular defect also occurs in vascular endothelium, which results in systemic inflammation and vascular occlusive complications, particularly in retinae and brain.

Murine *Ikbkg* deficiency is embryonic lethal on day d12 as a result of hepatic necrosis (53). In humans, hemizygosity for amorphic *IKBKG* alleles is usually lethal as well; IP is therefore almost always an XLD disorder of females, who are mosaic for cells lacking functional NEMO. In some cases, hyperpigmented whorls form that map to lines of Blaschko, reflecting migration of cells carrying the defect from the neural crest (54). Phenotypic heterogeneity is attributed to skewed X inactivation, and the propensity for selection against cells in which the normal X chromosome has been inactivated (55). Furthermore, evolution of the phenotype with age reflects selection against cells expressing the defective allele. Typically, these become less prominent during infancy (56). Similar mechanisms account for rare instances of in IP males, where it usually made compatible with survival by X-polyploidy (XXY) or somatic mosaicism (57, 58).

**Figure 3:**
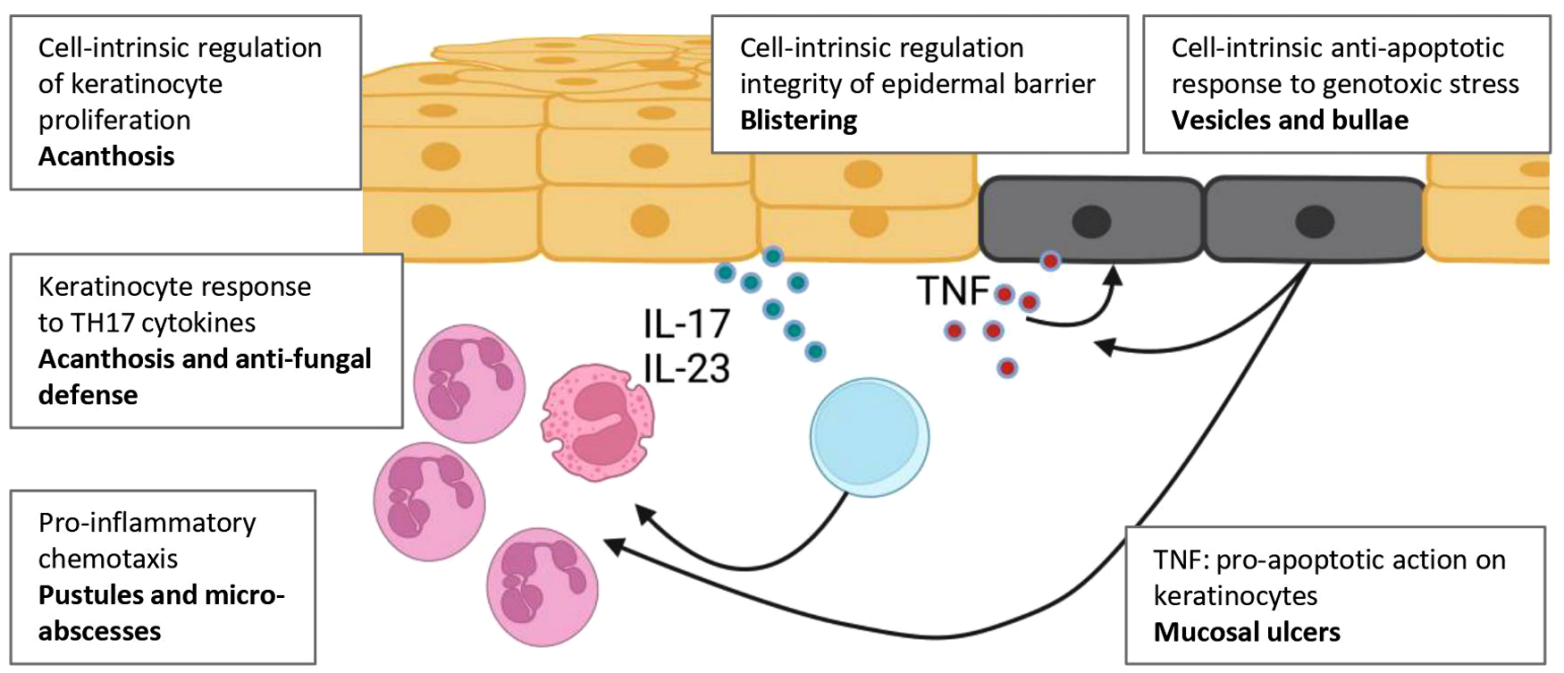
Overview of skin pathologies in which NF-κB is thought to contribute. NF-κB acts cell-intrinsically within keratinocytes, haemopoietic cells and other cells of the dermis, including fibroblasts (not shown). Cell-intrinsic defects in keratinocytes affect their proliferative capacity, propensity to undergo apoptosis, and capacity to contribute to the local antimicrobial response. NF-κB signalling also mediates (at least in part) keratinocytes responses to exogenous stimuli including pro-inflammatory cytokines (TNF, IL-1β) and Th17 cytokines. Keratinocyte activation and apoptosis can stimulate production of these cytokines. Created with BioRender.com.

In stages I and II of IP there is inflammation with epidermal oedema (spongiosis), epidermal blisters, and dermal cellular infiltrates, which progresses to apoptosis of mutant keratinocytes and skin atrophy with loss of skin appendages in stages III and IV (59). NEMO-deficient cells are more susceptible to undergoing apoptosis under the influence of pro-inflammatory cytokines TNF, IL1β (60) and genotoxic stress (61). During apoptosis, they release mediators including HSPs that ligate TLRs on and nearby cells with intact IKK complex. This triggers an inflammatory response and infiltrate (62), including release of proteases that disrupt desmosomes to cause blisters (Figure 3). In other words, there is a cycle of inflammation and apoptosis that continues until IKK-deficient cells are eliminated. These mechanisms may provide clues to the pathophysiology of rarer and more recently described NF-κB genodermatoses.

#### 3.2.2. *IKBKG* (NEMO): anhidrotic ectodermal dysplasia with immune deficiency (AED-ID)

IP is allelic with AED-ID, which results from hypomorphic *IKBKG* mutations. Features of AED-ID include hypohidrosis (sparse sweat glands), hypotrichosis (sparse hair), and periorbital wrinkling (63) (Table 3). Xerotic skin may take the form of severe ichthyotic like lesions. Various other cutaneous manifestations occur, including oral and perineal ulcers, palmoplantar keratoderma, hypopigmented papules seborrheic dermatitis, prurigo, and erythroderma, which may be severe (69).

**Table 3:** Links to images of conditions discussed.

Condition	URL	References
Incontinetia pigmenti (*IKBKG*)	https://edsociety.co.uk/what-is-ed/types-of-ed/welcome-to-the-incontinentia-pigmenti-section/	(64)
	https://www.nfed.org/learn/types/incontinentia-pigmenti/	
	https://dermnetnz.org/topics/incontinentia-pigmenti	
Mouth and genital ulcers (*TNFAIP3, RELA*)	https://dermnetnz.org/topics/behcet-disease	(65)
Pityriasis rubra pilaris (*CARD14*)	https://www.msdmanuals.com/professional/SearchResults?query = rubra + pilaris	(66)
Cylindromatosis (*CYLD*)	https://ijdvl.com/brooke-spiegler-syndrome-2/	(67)
Popliteal-pterygium syndrome (*CHUK, RIP4K*)	https://europepmc.org/article/med/22197489	(68)

Extracutaneous manifestations can include hypodontia, delayed tooth eruption and abnormal tooth shape (conical, accessory cusps, tulip-shaped teeth, and microdontia), low nasal bridge, frontal bossing and hypoplastic nasal alae (49, 69, 70). Severely affected individuals may show osteopetrosis and lymphedema (71). Isolated phenotypic features can phenotypes arise from defects in receptors responding to cues in cells that also signal though IKK (72, 73). At the other end of the spectrum, *IKBKG* mutations can present with mucocutaneous phenotypes of ichthyosis, eczematous dermatitis, palmoplantar keratoderma and recurrent mucocutaneous ulcers (69).

Immunodeficiency is a frequent but not invariable feature of hypomorphic *IKBKG* mutations. Indeed, some patients present with immunodeficiency in the absence of AED (74). Many patients are hypogammaglobulinaemic for IgG, sometimes with elevated IgM (63, 75–78). Most patients exhibit impaired polysaccharide-specific antibody responses (76) and some have NK cell defects (79). Pyogenic bacterial infections typically occur early in life, and later MSMD (80, 81). The reason for different immunological phenotypes remains to be resolved with certainty (8).

A large structural LoF variant in *IKBKG* (*Δ*exons4–10) accounts for about 80% of IP (82). More than 100 hypomorphic mutations in *IKBKG* have been described with considerable phenotypic variation (83, 84). Mutations affecting leucine zipper domain or N-terminal coiled-coil domains have a less severe clinical phenotype and may have isolated hypotonia (Mild AED-ID) (84). Mutations causing decreased protein expression, folding or stability can cause immunodeficiency without AED (74). Genetic diagnosis is problematic because a non-functional partial second copy of the *IKBKG* gene (pseudogene) is located distal to exon 10 thus making traditional PCR based molecular diagnostics tests difficult (85).

#### 3.2.3. *NFKBIA* (IκBα)

Rare cases of AED-ID have been observed in individuals with heterozygous defects in *NFKBIA* that confers defective phosphorylation and, therefore, resistance to IKK-mediated NF-κB activation (79, 86). The clinical phenotype of the index case was severe recurrent pyogenic bacterial infection, with low IgG and high IgM. T cells were refractory to activation. There were features of mild ED, with sparse hair, rough skin and conical teeth.

#### 3.2.4. *CHUK* (IKKα)

Homozygous LoF mutations in *CHUK* have been reported in patients with phenotypes on the popliteal-pterygia spectrum. Homozygous LoF mutations were reported in two fetuses with cocoon syndrome characterised by embryonic encasement with immobile limbs, omphalocele and craniofacial defects (29). Another child was reported with Bartocas-Papas syndrome, including alopecia totalis, microphthalmia, ankyloblepharon, cleft palate and popliteal webs (87) (Table 3). Another case arising from heterozygous *de novo CHUK* mutations was identified in a patient with ankyloblepharon-ectodermal defects-cleft lip/palate (AEC syndrome), as well as buccal synechiae, hypoplastic thumbs and 3rd-5th toe syndactyly (88). Ectodermal defects included sparse hair, conical teeth and nail defects. A patient with a similar phenotype was noted to by compound heterozygous mutations for a frameshift *CHUK* mutation transmitted from the unaffected mother and a *de novo* missense mutation (89). The latter two cases were also noted to have recurrent chest infections and hypogammaglobulinaemia.

It is interesting that immune deficiency has been observed in most of the cases that were live born. Bainter and colleagues reported a patient with homozygous missense *CHUK* mutation, and combined immune deficiency with total B-cell lymphopenia, panhypogammaglobulinaemia, and a progressive T-cell lymphopenia (90). Complications included recurrent pneumonia, failure to thrive, impetigo (*S. aureus*), mucocutaneous candidiasis, and epidermodysplasia verruciformis from HPV + . The child succumbed to overwhelming viraemia. As noted above, mouse models suggest that IKKα deficiency confers abnormalities in skin differentiation independently of NF-κB. In this case, the amino acid substitution was shown to prevent IKKα binding to NIK, and a defect in p100 processing was demonstrated in patient-derived fibroblasts but it remains unclear whether the skin manifestations were NF-κB-dependent. An engineered mouse model of this patient provided some insights into these manifestations, with a defect in cutaneous IL17A expression, absent lymph nodes and disorganised splenic architecture.

#### 3.2.5. *IKBKB* (IKKβ)

IKKβ deficiency results in severe combined immunodeficiency (SCID) with an inflammatory skin phenotype. The initial report described patients with a severe combined immune deficiency (SCID) phenotype complicated by disseminated mycobacteria (BCGosis, and *M. avium*), candidiasis, and recurrent gram negative infections (91). Dissemination of BCG to the skin resulting in a generalised rash has been described, and that case also featured ectodermal dysplasia (92).

Individuals have also been described with heterozygosity GoF missense *IKBKB* mutations (93, 94). The probands presented with recurrent respiratory infections/otitis media, cutaneous abscesses, bronchiectasis, ectodermal dysplasia, and lymphopenia, plus features of mild ED including abnormal dentition, nail defects, early onset cataracts and anhidrosis (93).

### 3.3. Canonical NF-κB defects

#### 3.3.1. *NFKB1* (p105/p50)

Heterozygous mutations in *NFKB1* encoding the precursor p105 results in p50 haploinsufficiency which appears to be the most common cause of Mendelian common variable immunodeficiency (CVID) (95). The associated clinical phenotypes are heterogenous, including a variety of autoinflammatory and rheumatological features (95–99) Autoimmune skin manifestations include vitiligo, alopecia, psoriasis and mucosal ulceration. Indeed, the phenotypic spectrum of NFKB1 haploinsufficiency appears to be broad and also encompasses mouth and genital ulceration, pyoderma gangrenosum, and erythema nodosum. In addition, skin infections such as bacterial abscesses, necrotizing cellulitis, folliculitis as well as viral infections have been observed (96, 100). It is associated with increased rates of lymphoproliferative disease, autoimmune haematological cytopenia, and enteropathies.

Immune dysregulation is observed in *Nfkb1*^−/−^ mouse models. Mice lacking p105 and p50 subunits display chronic inflammation, telomere shortening and cellular senescence associated with premature ageing (101, 102). *Nfkb1*^−/−^ mice display multi-organ autoimmunity with increased IL-6 production and activation of autoreactive CD8+ T cells (103, 104). *Nfkb1*^−/−^ mice are reported to display late onset al.opecia. A similar human phenotype was observed in New Zealand family with p50 haploinsufficiency (99).

#### 3.3.2. *RELA* (RelA, p65)

Haploinsufficiency for *RELA* has been reported to present with mouth and genital ulcers, recurrent fever, colitis and mucocutaneous ulceration (105, 106). Ulceration was attributed to heightened sensitivity to the apoptotic effects of TNF. Consistent with this, *Rela*^+/−^ mice demonstrated impaired NF-κB activation and develop cutaneous ulceration from TNF exposure.

#### 3.3.3. *REL* (c-Rel)

Homozygous hypomorphic *REL* deficiency has been reported in a patient with combined immunodeficiency and susceptibility to intracellular pathogens, sclerosing cholangitis, but no features of ED were described (107).

### 3.4. Non-canonical NF-κB defects

#### 3.4.1. *NFKB2* (p100/p52)

Human pathogenic variants in *NFKB2* were identified independently by several groups (108–110). Most patients were identified within cohorts of primary antibody deficiency patients, although they also suffer with organ-specific autoimmunity including alopecia areata, pneumonitis, autoimmune hepatitis, and arthritis (108, 111). Almost all *NFKB2* mutations described to date in patients with autoimmunity are missense and frameshift mutations located in exons 22 and 23 and affect the C-terminal portion of the protein that is responsible for regulating p100 processing (111).

Analysis of engineered mouse models has revealed that autoimmunity is T-cell dependent, and arises largely due to T cell-extrinsic actions of non-processible p100, which as described above, has IκB activity (112). In this case, p100 accumulation confers defects in thymic negative selection of conventional autoreactive T cells, and also impairs thymic selection of Tregs. This dominant GoF action of p100 stands in contrast with *NFKB1* variants, which mostly confer haploinsufficiency and therefore halve the abundance of p105/p50, and reveals that central T cell tolerance appears to be exquisitely sensitive to the IκB action of p100 (112). It remains to be determined with ED accounts for skin or pituitary defects.

#### 3.4.2. *RELB* (RelB)

Murine models of RelB deficiency have also been shown to develop an inflammatory skin phenotype similar to human atopic dermatitis (Barton et al., 2000). Autosomal recessive RelB deficiency have been found in family of patients presenting with combined immunodeficiency, autoimmune skin diseases and failure to thrive (Sharfe et al., 2015).

#### 3.4.3. *MAP3K14* (NIK)

Rare NIK deficiency arising from biallelic mutations in *MAP3K14* have been reported. Patients presented with recurrent viral, bacterial, and cryptosporidium infections (113). Immunological assessment identified B-cell lymphopenia, impaired class-switch recombination and somatic hypermutation, decreased marginal zone and memory B cells, and hypogammaglobulinaemia. No skin phenotype was described.

### 3.5. Upstream defects

#### 3.5.1. CBM complex

Four mammalian protein paralogs (CARD9, CARD10, CARD11 and CARD14) are characterized by presence of both CARD and coiled-coil domains operate in different tissues though a similar mechanism of recruiting BCL10 and MALT into a so-called CBM complex. The CBM complex regulates NF-κB, as well as other biochemical processes including mTORC1 activation (114, 115). CARD10 and CARD14 are expressed in keratinocytes, while CARD11 is expressed in lymphocytes and masts cells, and CARD9 is expressed in dendritic cells and neutrophils (116).

Human deficiencies of different CARD9, 11 and 14 have been described, and confer different skin phenotypes, although with some interesting shared features (117–122). Loss of function variants of *CARD11* have been identified in young children with SCID-phenotype. Complete deficiency of BCL10 and MALT1 have also been associated with combined immunodeficiency (123–128). MALT1 deficiency has also been associated with vitiligo, eczema, and erythroderma resembling Omenn syndrome (124–126, 129, 130). Mouse models suggest this is due to reduced Tregs (131).

Interestingly, hypomorphic mutations (dominant negative or haploinsufficiency) result in a complex phenotype of immune dysregulation that encompasses atopy, including atopic dermatitis and food allergy, and sometimes features of immune deficiency and autoimmunity (119–121, 132–134). Consistent with the proposition that hypomorphic rather than LoF mutations confer a skin phenotype, partial rescue of CARD11 function *via* somatic reversion of a LoF mutation has been reported to result in acquisition of an Omenn-like syndrome including hyper IgE and eczema (135). The mutations exhibit incomplete penetrance for AD. Similarly, atopic dermatitis (AD) plus hyper-IgE was observed with incomplete penetrance in one hypomorphic *Card11* hypomorphic mouse strain, while another manifested only late-onset hyper IgE (136–138). Neither phenotype was observed with complete *Card11* deficiency (139). In both humans and their mouse models, reduced CARD11 activity results in Th2-skewed immune responses, although there is evidence for both cell-intrinsic mechanisms, and cell-extrinsic effects *via* deficiency of Tregs (137, 140).

CARD14 expression is greatest in epithelia, particularly in the skin and respiratory tract. In both humans and mice, GoF *CARD14* mutations have been associated with psoriasis, or psoriatic-like syndromes of pityriasis rubra pilaris and palmoplantar pustulosis (66, 141, 142). Like dominant negative *CARD11* mutations, *CARD14* mutations that attenuate NF-κB activity are associated with AD and hyper-IgE (122), whereas complete absence of CARD14 is not associated with AD (141).

Intact CBM complexes are required for normal host defence, particularly at epithelial surfaces. Loss of function mutations in *CARD9* have been linked to autosomal recessive forms of susceptibility to chronic mucocutaneous candidiasis, deep dermatophytosis, and other infections involving yeast-like fungi (117), which appears to result from defective neutrophil recruitment and Th17 induction after ligation of dectin1 and dectin2 by fungal antigens.

#### 3.5.2. RIPK4

RIPK4 (receptor interacting serine-threonine kinase) interacts with PKC*δ* and mediates activation of NF-κB (143). RIPK4 and is critical for regulation of keratinocyte differentiation as well as craniofacial and limb development (144). The kinase domain of RIP4 activates NF-κB signalling during epidermal inflammatory responses (145). Loss of function mutations in *RIPK4* have been identified in patients with a popliteal-pterygium syndrome (Bartsocas-Papas syndrome) characterised by numerous craniofacial, musculoskeletal, genitourinary, gastrointestinal, cardiac and neurodevelopmental defects, resembling IKKα deficiency (68, 146, 147). Similar severe morphological defects and skin abnormalities of ectodermal origin can be seen in *CHUK* (IKKα) deficiency, as described above.

### 3.6. Defects of NF-κB regulation

#### 3.6.1. *TNFAIP3* (A20)

Haploinsufficiency for *TNFAIP3* results in an auto-inflammatory phenotype known also as Familial Behçet-like Autoinflammatory Syndrome or HA20 (65, 148). Reduction in A20 results in increased activity of canonical NF-κB, c-Jun N-terminal kinase, and p38. HA20 usually presents in children <10 years of age with recurrent oral and genital ulcers. Skin involvement is noted in approximately 50% of cases, with pathergy, pustules, folliculitis and acneiform eruptions (Table 3). Additional manifestations include periodic fever, recurrent infections, ocular inflammation, gastrointestinal symptoms ranging from pain to inflammatory disease with perforation, and polyarthropathy. Some patients exhibit frank immune deficiency or organ-specific autoimmunity (149, 150).

#### 3.6.2. CYLD

Autosomal dominant mutations in *CYLD* cause familial cylindromatosis characterised by multiple benign skin tumours (cylindromas) (151, 152). CYLD is expressed at highest levels in brain, testis, skin and thymus (153). CYLD is a deubiquitinase that targets several proteins, including NEMO, TRAF2, TRAF6, BCL3, and TRAF2 (154). CYLD is therefore a negative regulator of NF-κB-mediated pro-survival genes, and an inhibitor of necroptosis mediated by RIPK1 after ligation of TLRs or TNFR1 (155–160). Mutations in CYLD account for what had previously been three skin appendage tumour syndromes, familial cylindromatosis, Spiegler-Brooke syndrome (BSS), and multiple familial trichoepithelioma. Cylindromas arising from hair follicles or sweat glands usually on the scalp or face. BSS consists of a triad of cylindromas, trichoepitheliomas arising from hair follicles usually affecting nose and nasolabial folds and spiradenomas arising from sweat glands typically on the head, neck and trunk (161) (Table 3). CYLD deficiency is not associated with overt immune deficiency. Although a *Cyld*^−/−^ mouse model exhibited a T cell developmental defect this was not observed in other models (162). These models also feature susceptibility to chemically-induced skin tumours induced colonic inflammation and increased incidence of tumors and enhanced NF-κB activity in lymphocytes and myeloid cells (162).

#### 3.6.3. OTULIN

(OTU Deubiquitinase With Linear Linkage Specificity) is a negative regulator of NF-κB. Homozygous deficiency of *OTULIN* results in OTULIN-related Autoinflammatory Syndrome (ORAS), which presents in infancy with neutrophlic nodular panniculitis, lipodystrophy and cutaneous vasculitis, as well as recurrent fevers, diarrhoea, and arthritis, and is characterised by prominent acute phase responses and hypergammaglobulinaemia (163–165). More recently, a different syndrome characterised by skin necrosis and necrotising pneumonia due to *S. aureus* has been identified in individuals with *OTULIN* haploinsufficiency (166). Patients often present in adolescence. The phenotype is thought to result in a cell-intrinsic susceptibility of fibroblasts to cytotoxic effects of staphylococcal α-toxin and appears to be mediated by accumulation of caveolin-1 rather than heightened NF-κB activity in response to TNF.

## 4. Conclusions

Cutaneous manifestations provide important clues to IEI arising from defects in NF-κB. The manifestations are diverse (Table 4; Figure 2). Disorders associated with prominent defects in ectodermal development include those arising from *IKBKG* and *NFKBIA* variants, manifesting as ED with loss of skin appendages, and popliteal-pterygium spectrum (Bartsocas-Papas syndrome), from defects in *CHUK* and *RIPK4*. By contrast, defects of regulatory proteins (A20, OTULIN) result in predominantly inflammatory manifestations of mucocutaneous ulceration and neutrophil accumulation in the skin, features that make these syndromes an important differential diagnosis for Behcet's disease. CYLD defects result in excessive expansion of keratinocytes and skin appendages (Figure 3).

**Table 4:** Summary of skin and immune phenotypes with NF-κB defects.

Gene	Protein	Inheritance	Type	NF-κB effect	Immune phenotype	Skin phenotype
*IKBKG*	NEMO	XLD	LoF	Loss of IKK activity	Normal (CID rarely)	Incontinentia pigmenti Vesicular bullous, hyperpigmentation whorls, then depigmentation, atrophy, oral and genital ulcers
		XLR	Hypomorphic	Reduced of IKK activity	Hypogammagloblinaemia Specific antibody deficiency MSMD	Anhidrotic ectodermal dysplasia Sparse sweat glands, sparse hair, periorbital wrinkles, palmoplantar keratoderma, erythroderma
*CHUK*	IKKα	AR	LoF	Loss of IKK activity (canonical and non-canonical)	Embryonic lethal	Cocoon syndrome
				Reduced IKK activity (canonical and non-canonical)	CID Hypogammaglobulinaemia CMC	Popliteal-pterygium spectrum (Bartocas-Papas syndrome) Alopecia Sparse hair Mucocutaneous candidiasis
*IKBKB*	IKKβ	AR	LoF	Loss of canonical IKK activity	SCID	Cutaneous opportunistic infection
		AD	Hypermorphic	Increased canonical IKK activity	CID	Mild ectodermal dysplasia Hidradenitis suppurativa Nail changes
*MAP3K14*	NIK	AR	LoF	Loss of non-canonical IKK activity	SCID	Nil
*NFKBIA*	IκBα	AR	Hypermorphic	Reduced of IKK activity (canonical and non-canonical)	Hypogammaglobulinaemia Pyogenic bacterial infection	Anhidrotic ectodermal dysplasia Sparse sweat glands, sparse hair
*NFKB1*	p105/p50	AD	Haploinsufficiency	Reduced of IKK activity (canonical and non-canonical)	Hypogammaglobulinaemia Autoimmunity	Vitiligo, alopecia, psoriasis, mucosal ulceration, pyoderma gangrenosum, erythema nodosum, infection
*REL*	c-Rel	AR	LoF	Reduced of IKK activity	CID	Nil
*NFKB2*	p100/p52	AD	Dominant negative p100 (IkB) action	Reduced of IKK activity (canonical and non-canonical)	Hypogammaglobulinaemia Autoimmunity	Alopecia
*RELA*	p65	AD	Haploinsufficiency	Reduced canonical IKK activity	CID	Mouth and genital ulcers
*RELB*	RelB	AR	LoF	Reduced non-canonical activity	CID	Atopic dermatitis
*CARD11*	CARD11	AR	LoF	Loss of canonical IKK activity	SCID	
		AD	DN/haploinsufficiency	Reduced canonical IKK activity	Hypogammaglobulinaemia	Atopic dermatitis
*MALT1*	MALT1	AR	LoF	Loss of canonical IKK activity	SCID	Erythroderma, vitiligo, atopic dermatitis
*BCL10*	BCL10	AR	LoF	Loss of canonical IKK activity	SCID	Nil
*CARD9*	CARD9	AR	LoF	Reduced canonical IKK activity	CMC	CMC, dermatophyte
*CARD14*	CARD14	AD	GoF	Increased canonical IKK activity	Nil	Pityriasis rubra pilaris Psoriasis
*CYLD*	AYLD	AR	LoF	Reduced of IKK activity (canonical and non-canonical)	Nil	Familial cylindromatosis, Spiegler-Brooke syndrome, multiple familial trichepithelioma
*TNFAIP3*	A20	AD	Haploinsufficiency	Increased canonical IKK activity	Autoinflammatory	Mucocutaneous ulceration, pathergy, pustules, acne
*OTULIN*	OTULIN	AR	LoF	Reduced of IKK activity (canonical and non-canonical)	Autoinflammatory	Neutrophilic panniculitis, lipodystrophy
*RIPK4*	RIPK4	AR	LoF	Loss of canonical IKK activity	Autoinflammatory	Popliteal-pterygium syndrome (Bartsocas-Papas syndrome)

AD, autosomal dominant; AR, autosomal recessive; CID, combined immune deficiency; CMC, chronic mucocutaneous candidiasis; DN, dominant negative; GoF, gain-of-function; LoF, loss-of-function; MSMD, Mendelian susceptibility to mycobacterial disease; SCID, severe combined immune deficiency.

**Figure 4:**
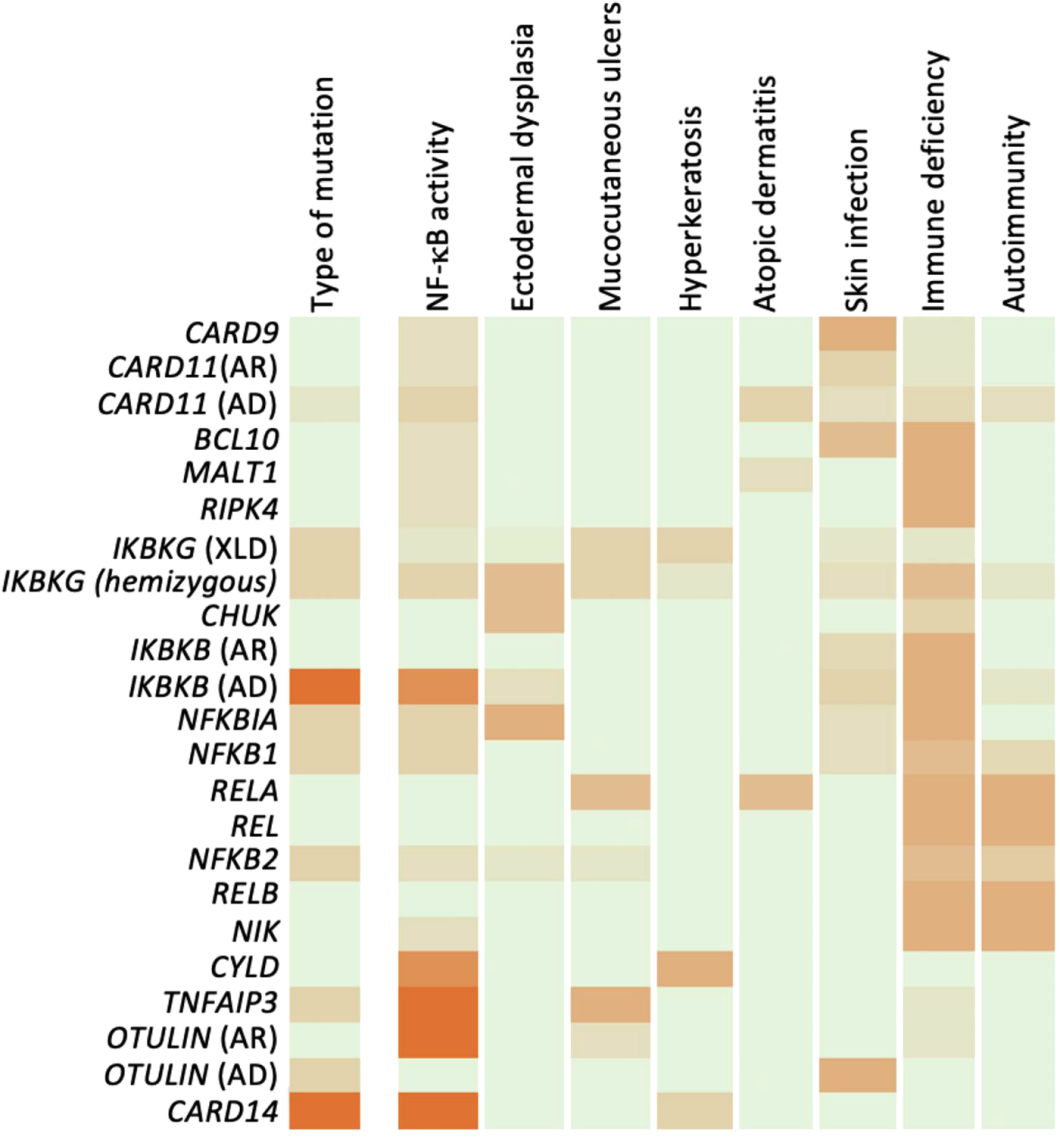
Genotype-phenotype relation of human NF-κB defects. Summary of mutations and phenotypes discussed, expressed as a heatmap. For genotype and NF-κB effect, reduced activity appears green and increased activity in orange. For phenotypes, absence of phenotype is indicated in white, while severity of phenotype is shown in orange.

It is still too early to arrive at a coherent account of all the genotype-phenotype relations for each of the IEIs described in this review. They nevertheless provide insights into pathogenic pathways likely to be relevant to more common disorders where it can be assumed that pathology arises from complex interactions of more than one gene defect (Figure 4). These include psoriasis, skin cancers, and syndromes associated with mucocutaneous ulceration such as Behcet's disease and inflammatory bowel disease.

Psoriasis is characterised by acanthosis, increased dermal vascularity, and neutrophilic microabscesses in the epidermis. IEIs provide evidence for the importance of NF-κB in regulating keratinocyte proliferation. Of the IEIs discussed here, GoF variants in *CARD14* account for segregation of psoriasis with the Psors2 (17q25-ter) locus on genome-wide linkage scans. Disease-associated alleles of *CARD14* result in increased NF-κB activity in keratinocytes in response to IL-17A (167). Furthermore, while some models have suggested pathogenic pathways common to psoriasis and atopic dermatitis, it is noteworthy that *CARD14* defects with the opposite polarity to those responsible for psoriasis (i.e., LoF alleles) cause severe atopic dermatitis (122). Furthermore, while excessive NF-κB signals triggered by Th17 cytokines appears to contribute to psoriatic changes, underactivity of this pathway may explain predilection to cutaneous yeast and fungal infections, as observed with hypomorphic defects in *CARD9*.

Both *CARD14* GoF and HA20 result in increased NF-κB activity. *CARD14* GoF results in both plaque and pustular psoriasis. The most consistent clinical feature of HA20 appears to be severe and early onset mouth and genital ulcers, nevertheless, palmoplantar pustules are also observed frequently (65), whereas plaque psoriasis does not appear to be a feature of HA20. HA20 is also a profoundly inflammatory disorder, and these differences probably reflect consequences of A20 deficiency within cells of the immune system. Further complexity, however, is revealed by the Behcet's disease-like presentations observed in association with *NFKB1* haploinsufficiency. Thus, attributing the aphthosis to NF-κB alone might be an oversimplification.

A number of variables are likely to account for the phenotypic variation from Mendelian defects in NF-κB, including the severity of the biochemical defect. As illustrated for *CARD11*, *OTULIN*, and *IKBKB*, autosomal dominant defects that confer quantitative changes in NF-κB signalling result in phenotypes that differ significantly from LoF mutations. Other explanations for phenotypic variation include the consequences of feed-forward loops, particularly for the autoinflammatory disorders (exemplified by HA20). In the interests of concision, we have concentrated on changes in NF-κB, but many of the molecules discussed also regulate other pathways. Whether or not this is the case, background genetic variation will of course also contribute to phenotypic variation.

Finally, as noted above, there are differences in cell-specific expression. Pathology in the skin is common with NF-κB disorders because it acts intrinsically in keratinocytes, as well as in inflammatory cells that infiltrate the epidermis. Expression of some of the regulators described in this review, however, are restricted to one compartment or another. Dissecting these mechanisms is likely to require accurate models of the defects in question, in which the genetic defects can be isolated to either keratinocytes or components of the immune system. The phenotypes observed with IEIs provide evidence that this line of enquiry is likely to be informative for more common disease.
